# Seawater pH Measurement: A Critical Metrological Assessment of Operational Scales and the Unified pH Scale

**DOI:** 10.3390/s26206514

**Published:** 2026-10-14

**Authors:** Raquel Quendera, Maria João Nunes, Ana Luísa Fernando, Carla Palma, Marta Serra Giza, Olivier Pellegrino, João Alves e Sousa

**Affiliations:** 1 Departamento de Metrologia, Instituto Português da Qualidade, R António Gião 2, 2829-513 Caparica, Portugal; 2 Chemistry Department, Nova School of Science and Technology|NOVA FCT, NOVA University of Lisbon, Campus Caparica, 2829-516 Caparica, Portugal; 3 LAQV REQUIMTE, Associated Laboratory for Green Chemistry, Chemistry Department, NOVA School of Science and Technology|NOVA FCT, NOVA University of Lisbon, Lisbon Campus, 2819-516 Caparica, Portugal; 4 Escola Superior de Tecnologia do Barreiro, Universidade Politécnica de Setúbal, R. Américo Silva Marinho, 2839-001 Lavradio, Portugal; 5 MEtRICs/CubicB, Chemistry Department, NOVA School of Science and Technology, NOVA FCT, NOVA University of Lisbon, Campus Caparica, 2829-516 Caparica, Portugal; 6 Instituto Hidrográfico, Rua das Trinas, 49, 1249-093 Lisboa, Portugal

**Keywords:** metrological traceability, ocean acidification, pH scales, sensors, unified pH scale

## Abstract

Ocean acidification, driven by rising anthropogenic CO_2_, causes a measurable decrease in seawater pH with consequences for marine ecosystems. Accurate, traceable pH measurement is essential for ocean monitoring sensors. This critical review examines five pH scales: the IUPAC activity scale (pH), the free proton scale (pH_F_), the total proton scale (pH_T_), the seawater scale (pH_SWS_), and the unified scale (pH_abs_). For each scale, the analytical methods, metrological assumptions, traceability chain to the SI, and measurement uncertainties are analyzed. The review updates the foundational 2016 work with subsequent developments and focuses particularly on the application of pH_abs_ to saline solutions. In its current differential-potentiometric realization, pH_abs_ yields an expanded uncertainty *U*(pH) ≈ 0.2 in seawater. GOA-ON expresses its goals as standard uncertainties (*k* = 1): 0.003 (climate) and 0.02 (weather), equivalent to *U* ≤ 0.006 and *U* ≤ 0.04 for *k* = 2. The reported *U*(pH) ≈ 0.2 is therefore approximately 33 times the climate-quality target and approximately 5 times the weather-quality target, compared at the same coverage. The dominant contribution is the residual liquid-junction potential; the standard uncertainty of 6.3 mV (*k* = 1) reported in dilute solutions corresponds, at 25 °C, to *u*(pH) ≈ 0.1 and *U*(pH) ≈ 0.2 for *k* = 2, consistent with the reported *U*(pH) ≈ 0.2. However, this dilute-solution estimate is not a validated uncertainty for seawater and should not be extrapolated to seawater without experimental validation. Liquid-junction effects are therefore a priority for potentiometric sensors and for the current pH_abs_ realization. Spectrophotometric pH_T_ sensors avoid a liquid junction but have distinct uncertainty contributions related to indicator characterization, optical measurement, temperature, salinity, and sample perturbation.

## 1. Introduction

The oceans play a fundamental role in the global carbon dioxide (CO_2_) cycle, absorbing approximately 25–30% of CO_2_ emissions and thereby acting as a critical buffer for Earth’s climate [1,2]. The increasing release of anthropogenic CO_2_ to the atmosphere is leading to ocean acidification with the pH of ocean surface water decreasing by approximately 0.1 units since pre-industrial times (from 8.2 to 8.1) [3,4]. Projections under high-emission scenarios suggest a further decrease of 0.2 and more by 2100 [5]. These changes alter the chemistry of the marine carbonate system, threatening calcifying organisms, disrupting food webs, and affecting ecosystem services of global importance [6,7].

**Figure 1:**
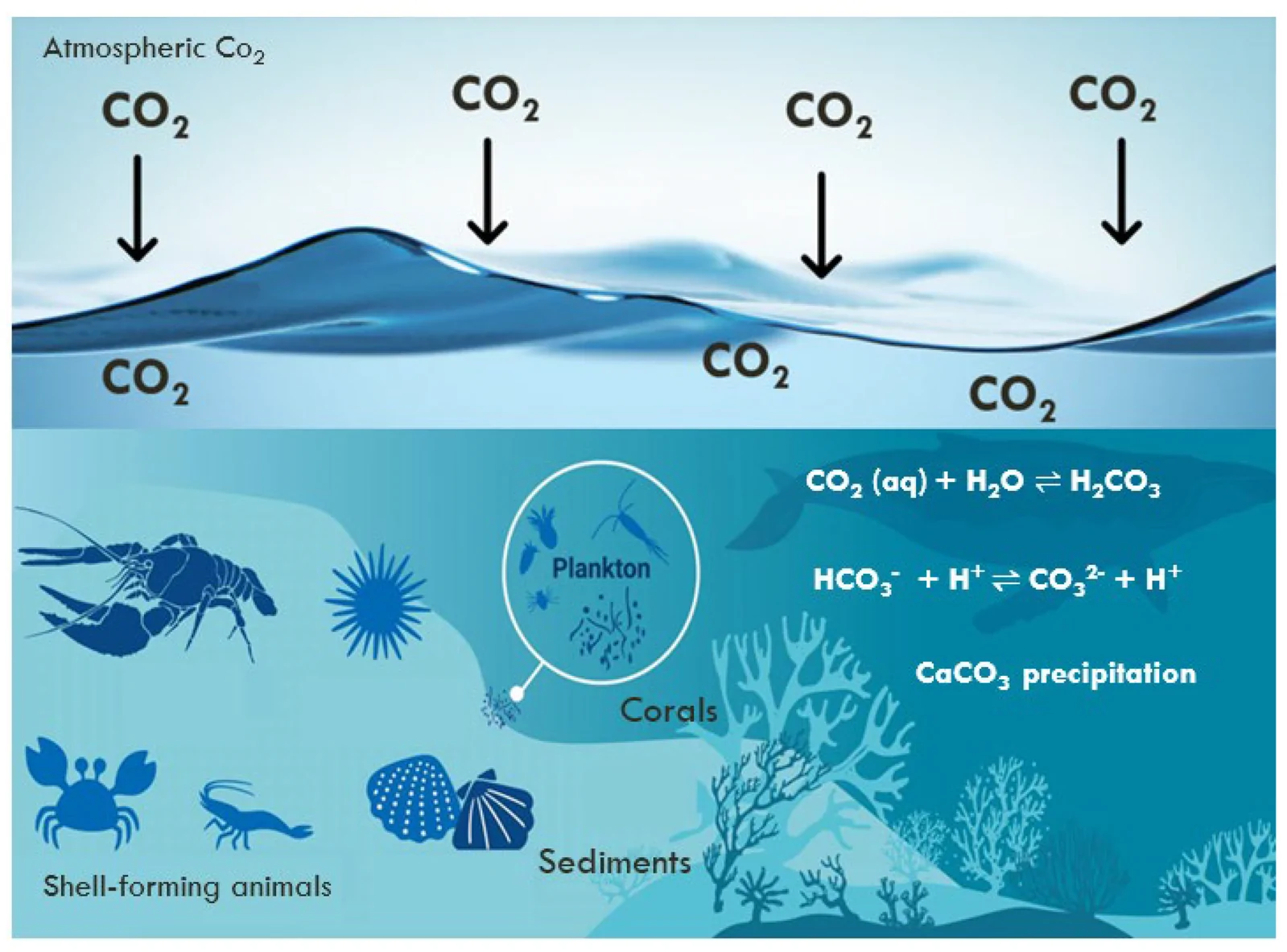
Schematic diagram of carbon dioxide system and the reactions with seawater (adapted from [10,11]).

pH quantifies acidity in aqueous, non-aqueous, and mixed media and is a frequently measured parameter in chemistry, biology, and oceanography. In seawater, pH is a function of the CO_2_ system and is essential for understanding physical and chemical ocean processes. Atmospheric CO_2_ dissolves in surface seawater. Subsequent hydration and carbonate-system equilibria increase the hydrogen-ion concentration and decrease the carbonate-ion concentration. These changes affect the saturation state and stability of calcium carbonate minerals such as calcite and aragonite [8,9,10,11]. Figure 1 illustrates atmospheric CO_2_ uptake by seawater and the resulting acid–base equilibria. The dissolved CO_2_ species are represented collectively by [CO_2_]*.

The following acid–base equilibria are formed and represented by Equations (1)–(3):

(1) $$K_{0}={\left[{\mathrm{CO}}_{2}\right]}^{*}/f\left({\mathrm{CO}}_{2}\right)$$

(2) $$K_{1}=\left[H^{+}\right]\left[{\mathrm{HCO}}_{3}^{-}\right]/{\left[{\mathrm{CO}}_{2}\right]}^{*}$$

(3) $$K_{2}=\left[H^{+}\right]\left[{\mathrm{CO}}_{3}^{2-}\right]∕\left[{\mathrm{HCO}}_{3}^{-}\right]$$

In Equation (1), *f*CO_2_ is the fugacity of CO_2_ in the gas phase: an effective pressure that expresses its chemical potential and accounts for non-ideal gas behaviour. It is not the concentration of CO_2_ in the aqueous phase. The solubility coefficient *K*_0_ relates *f*CO_2_ to dissolved [CO_2_]* at the specified temperature, salinity, and pressure. In Equations (2) and (3), *K*_1_ and *K*_2_ are the first and second acid-dissociation constants of the carbonate system, respectively. Square brackets denote the amount content in mol·kg^−1^ of solution [8,10].

The boron element (B) is present in seawater mainly in the form of boric acid (H_3_BO_3_), a weak acid. Despite its minor concentration compared to sodium (Na^+^), chloride (Cl^−^), magnesium (Mg^2+^) and calcium (Ca^2+^) ions, it is a paramount element contributing to the buffering capacity of seawater, to the alkalinity and to the maintenance of life in the marine ecosystem. In this context, the dissociation of boric acid and the self-ionization of water are acid–base equilibria that need to be considered in seawater composition [8,10,12].

(4) $$K_{B}=\left[H^{+}\right]\left[B{\left(\mathrm{OH}\right)}_{4}^{-}\right]/\left[B{\left(\mathrm{OH}\right)}_{3}\right]$$

*K*_W_ = [H^+^][OH^−^]. (5)

In Equation (4), *K*_B_ is the dissociation constant of H_3_BO_3_ and in Equation (5) *K*_W_ is the equilibrium constant for the self-ionization of water representing the equilibrium between H^+^ and the hydroxide ions (OH^−^).

The buffering capacity of seawater is essential for sustaining life in marine ecosystems, as it helps to stabilize and minimize pH fluctuations. The concentration of carbon species (dissolved inorganic carbon) in seawater is significantly higher than the [H^+^] ions produced. As a result, the addition or removal of carbon species is balanced by their conversion between different forms, leading to minimal changes in [H^+^] [13,14].

As anthropogenic [CO_2_] increases in the atmosphere, additional CO_2_ is absorbed by surface seawater. Carbonate equilibria then increase [H^+^] and decrease [CO_3_^2−^], diminishing the ocean’s buffering capacity.

As mentioned before, prior to the advent of industrialization, the ocean’s surface had an average pH of approximately 8.2. However, due to human activities such as fossil fuel combustion and deforestation, it is now estimated to have declined to around 8.1 [2,3,5].

Seawater is a saline medium containing macronutrients such as phosphorus, nitrogen, and silicon, as well as trace metals such as copper, cadmium, cobalt, iron, manganese, nickel, and zinc. Although present in trace concentrations, these metals are vital to marine life, playing an essential role in the regulation of biological and biochemical processes in seawater. Changes in the ocean’s state and chemistry, particularly a decrease in pH, can have detrimental effects on the marine ecosystem that have not yet been fully predicted [1,3]. One possible effect is the chemical speciation of metals in seawater, where essential metals, such as cadmium and copper, can become harmful to the marine system [15,16]. As stated above, the increase in atmospheric CO_2_ decreases the pH of the ocean, resulting in a decrease in the [OH^−^] and [CO_3_^2−^] ions. As the pH of seawater decreases, these anions become less available to complex with metals, resulting in a higher concentration of free metals [1,17]. Toxic heavy metals, such as arsenic, lead, mercury, and chromium, can be transferred to living organisms, leading to contamination and accumulation in food chains, with harmful effects on all ecosystems [18]. Another consequence of ocean acidification is the potential extinction of shell-forming organisms. Marine species from coastal regions, such as mussels, clams, and oysters, rely on the carbonate system to form their shells and skeletons. As the availability of this mineral decreases, these organisms will have more difficulty synthesizing it, expending too much energy in the process and consuming the energy that would be necessary to feed themselves. This can consequently lead to their extinction. Open ocean species, such as foraminifers, coccolithophores, and pteropods, are directly affected by acidification and may also disappear. As the pH of the ocean decreases, the species at the basis of the marine food chain, particularly phytoplankton, can decline in number or disappear. This will also affect the organisms that feed on them, disrupting the human food chain. The extinction of organisms such as corals, which are nesting areas for species of commercial interest, could put marine biodiversity at risk, affecting human activities such as tourism and fishing [6,7].

Accurate seawater pH measurement is therefore not merely an analytical problem but a scientific and societal priority. GOA-ON defines two quality goals, both expressed as standard uncertainties (*k* = 1): a climate-quality goal of *u* ≤ 0.003 pH units for detecting long-term trends and a weather-quality goal of *u* ≤ 0.02 pH units for resolving larger spatial and temporal variability [19,20]. In this review, *u* denotes standard uncertainty and *U* = *ku* denotes expanded uncertainty. For *k* = 2 (approximately 95% confidence level for a Gaussian distribution) the two goals correspond to *U* ≤ 0.006 and *U* ≤ 0.04, respectively; these are the same goals expressed at a different coverage, not additional quality categories. Comparisons between performance and quality objectives specify whether standard or expanded uncertainty is used. This need for harmonization is underscored by United Nations Sustainable Development Goal 14.3, whose indicator 14.3.1 requires primary-quality data with quantified measurement uncertainty [21,22,23]. Seawater is a complex high-ionic-strength medium (*I* ≥ 0.7 mol·kg^−1^), so pH techniques and traceability chains developed for dilute aqueous solutions cannot be transferred without additional assumptions and uncertainty contributions.

A comprehensive review of operational seawater pH scales and their metrological basis was published by Dickson et al. in 2016 [24], and remains the key reference for the field. That review, however, pre-dates: (i) the extension of chemical-speciation (Pitzer-based) models to TRIS buffers across wider temperature and salinity ranges [25,26,27]; (ii) the characterization of spectrophotometric indicator dyes and quantification of impurity effects on pH_T_ [28,29]; (iii) metrological work on the traceability chain and uncertainty budget for the Harned-cell primary method using TRIS buffers of different salinities [22,23,30,31,32]; and, most significantly, (iv) the introduction and first experimental realization of the unified pH scale (pH_abs_), a fundamentally different, chemical-potential-based approach to expressing acidity that has been proposed as a media-independent alternative to activity and concentration-based scales [33,34,35,36,37,38,39,40].

The present paper does not attempt to replace the 2016 review, whose treatment of the classical scales remains authoritative; instead, it (a) updates the classical-scale discussion with the post-2016 literature, (b) reframes the comparison explicitly around the sensing principle used to realize each scale, spectrophotometric indicator-dye sensors, and the differential-potentiometry technique used for pH_abs_ and (c) provides a critical, rather than descriptive evaluation of the unified pH scale, which to date has not been assessed against the practical performance requirements of seawater monitoring.

This article is a critical narrative review rather than a systematic review or meta-analysis. The literature was identified through targeted searches of Web of Science, Scopus, Crossref, and publisher databases using combinations of the terms seawater pH, total pH scale, free pH scale, seawater pH scale, unified pH, absolute pH, TRIS buffer, Harned cell, liquid-junction potential, spectrophotometric pH, ISFET, optode, and ocean-acidification sensor. Citation chaining from the 2016 metrological review and key technical reports was also used. Priority was given to IUPAC and metrology guidance, primary-method and reference-material studies, uncertainty evaluations, indicator-characterization studies, and peer-reviewed demonstrations in saline matrices. Studies were included when they defined or realized a scale, quantified an uncertainty contribution, characterized a seawater sensor, or evaluated performance under saline conditions. Non-seawater applications without a transferable metrological or sensing contribution were excluded. No preregistered protocol, exhaustive screening log, or risk-of-bias assessment was applied.

The remainder of this paper is organized as follows. Section 2 summarizes the physicochemical basis, sensing principles, and traceability chains of the pH scales considered, culminating in an assessment of the current experimental realization of pH_abs_ in saline solutions (Section 2.3.2). Section 3 compares pH scales and sensing platforms in terms of metrological performance and practical suitability for seawater monitoring. Section 4 discusses their principal metrological challenges and the supporting role of pH in emerging-pollutant monitoring. Section 5 presents the conclusions and priorities for sensor development, including the control of liquid-junction effects in potentiometric methods and of indicator, optical, and sample-perturbation effects in spectrophotometric methods.

### Measurable Analytical Parameters of the CO_2_ System

The ocean carbonate system can be fully described by four measurable parameters: *p*CO_2_, dissolved inorganic carbon (DIC), total alkalinity (TA), and pH. Measuring any two of these, combined with known thermodynamic constants as salinity (*S*), temperature (*T*), and pressure (*P*), allows the remaining variables to be calculated [9,10]. TA and DIC are independent of *P* and *T* and are among the most frequently measured parameters; on the other hand, pH and *p*CO_2_ are sensitive to both [41]. Compared with other carbonate system variables, seawater pH data suffers from insufficient metrological consistency between different measurement approaches and between measured and calculated values. Because of this, the analytical quality of pH sensing has a direct and amplified effect on the accuracy of the whole carbonate-system characterization [42,43].

## 2. Operational pH Scales: Methods and Sensing Principles

Each pH scale described in this section—namely the IUPAC pH scale or activity-based scale (pH), the free proton pH scale (pH_F_), the total proton pH scale (pH_T_), the seawater pH scale (pH_SWS_) and finally the unified pH scale (pH_abs_)—has a specific thermodynamic meaning, an associated analytical method, and a defined set of assumptions. These assumptions determine the scale’s uncertainty budget and the conditions under which it is valid. Conflating scales, or applying a scale outside its domain of validity, introduces systematic errors that are difficult to detect and quantify.

### 2.1. The pH Scale

This pH scale, formalized by Buck et al. [44], is defined as the negative decimal logarithm of the molal activity of H^+^, as shown in Equation (6).

(6) $$\mathrm{pH}=-\mathrm{lg}a_{H^{+}}=-\mathrm{lg}\left(\frac{b_{H^{+}}\gamma_{H^{+}}}{b^{0}}\right),$$

where *b*_H+_ (mol·kg^−1^) and *γ*_H+_ are the molality and molal activity coefficient of H^+^, respectively, and *b*^0^ = 1 mol·kg^−1^ is the standard molality. Because the single-ion activity *a*_H+_ cannot be determined by any thermodynamically rigorous method, pH is experimentally assigned using the primary potentiometric method with the Harned cell (a cell without a liquid junction) with the following configuration [22,23,30,44,45,46]:

Pt | H_2_ (g, 1 atm) | Buffer solution with Cl^−^ |AgCl | Ag, (Cell I)

where Pt/H_2_ is the standard gaseous hydrogen electrode (SHE) with a standard potential (*E*^0^ = 0 V) and Ag/AgCl is the silver/silver chloride reference electrode.

The potential of cell (I) is described by the Nernst equation, as expressed in Equation (7).

(7) $$E=E_{\mathrm{Ag},\mathrm{AgCl}}^{0}-\left(\frac{\mathrm{RT}\ln\left(10\right)}{F}\right)\mathrm{lg}\left[\left(\frac{b_{H}\gamma_{H}}{b^{0}}\right)\cdot \left(\frac{b_{\mathrm{Cl}}\gamma_{\mathrm{Cl}}}{b^{0}}\right)\right],$$

where *E* (V) is the potential difference measured experimentally in a standard buffer solution and *E*^0^_Ag,AgCl_ (V) is the standard potential of the Ag/AgCl electrodes. All the potential measurements are corrected to a hydrogen partial pressure of 101325 Pa. *R*, *F* and *T* are the molar gas constant (8.3144626 J mol^−1^·K^−1^), the Faraday constant (96,485.33212 C·mol^−1^) and the absolute temperature in K, respectively. *b*_H_ and *b*_Cl_ are the molal concentrations (mol·kg^−1^) of H^+^ and Cl^−^ and *γ*_H_ and *γ*_Cl_ are the respective molal activity coefficients.

The Ag/AgCl electrodes are not commercially available and their *E*^0^_Ag,AgCl_ needs to be experimentally determined in a 0.01 mol·kg^−1^ HCl solution using the Harned cell in the following configuration:

Pt | H_2_ (g, 1 atm) | HCl (*b*) | AgCl | Ag, (Cell Ia)

According to the Nernst Equation, the potential of cell (Ia) is given by Equation (8) [44].

(8) $$E_{\mathrm{Ag},\mathrm{AgCl}}^{0}=E_{\mathrm{HCl}}+\left(2\frac{\mathrm{RT}\ln(10)}{F}\right)\mathrm{lg}\left[\left(\frac{b_{\mathrm{HCl}}}{b^{0}}\right)\left(\gamma_{\mathrm{HCl}}^{\pm }\right)\right],$$

where *E*_HCl_ (V) is the potential difference measured in the 0.01 mol·kg^−1^ HCl solution and *γ^±^*_HCl_ is the mean ionic activity coefficient of HCl (mol·kg^−1^).

Substituting *b*_H_*γ*_H_ = *a*_H_ into Equation (7) and rearranging gives:

(9) $$-\mathrm{lg}\left(\frac{a_{H}\gamma_{\mathrm{Cl}}}{b^{0}}\right)=\frac{\left(E-E_{\mathrm{Ag},\mathrm{AgCl}}^{0}\right)F}{\mathrm{RT}\ln\left(10\right)}+\left[\mathrm{lg}\left(\frac{b_{\mathrm{Cl}}}{b^{0}}\right)\right].$$

The term on the left-hand side of Equation (9) is the acidity function p(*a*_H_*γ*_Cl_) which is represented as a function of *b*_Cl_^−^ and is extrapolated to the zero to obtain the standard acidity function p(*a*_H_*γ*_Cl_ )^0^. The pH of primary buffers is then assigned according to Equation (10).

(10) $$\mathrm{pH}=p{\left(a_{H}\gamma_{\mathrm{Cl}}\right)}^{0}+\mathrm{lg}\left(\gamma_{\mathrm{Cl}}\right),$$

and *γ*_Cl_ is then estimated using the Bates–Guggenheim convention as expressed in Equation (11).

(11) $$\mathrm{lg}\left(\gamma_{\mathrm{Cl}}\right)=-\frac{{\mathrm{AI}}^{1/2}}{1+1.5I^{1/2}},$$

where *A* is the Debye–Hückel coefficient and *I* (mol·kg^−1^) is the ionic strength of the solution.

In the Harned cell method, each step has a well-defined mathematical model and associated standard uncertainties, with low expanded uncertainty (*U* ≤ 0.004).

The final result is only traceable to the International System of Units (SI) if the *U* associated with the Bates–Guggenheim convention for calculating lg(*γ*_Cl_) is estimated as 0.01 (*k* = 2) [44,47,48].

**Figure 2:**
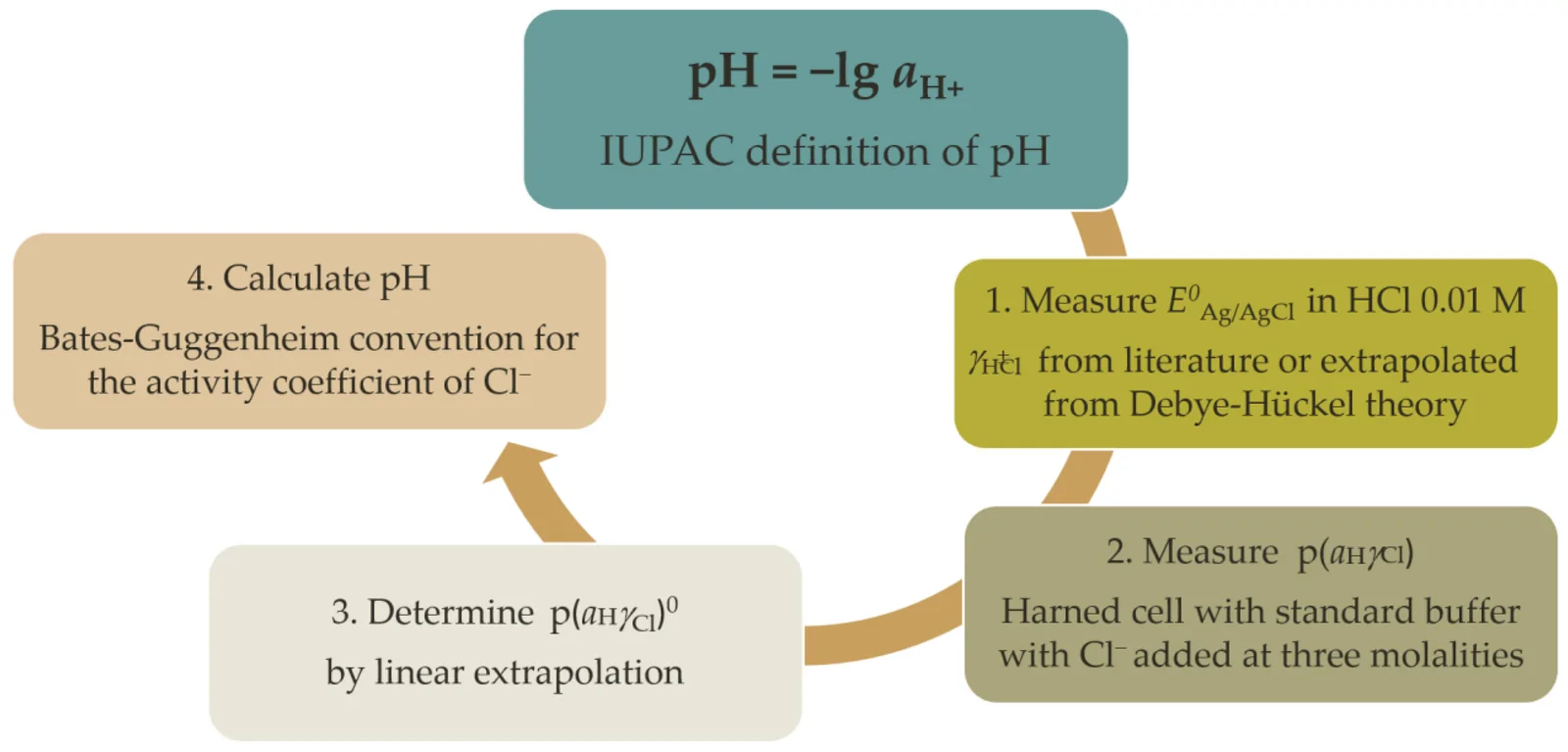
Essential steps in using the Harned cell to assign pH values to primary buffers (adapted from [44]).

Figure 2 summarizes the necessary operations with the Harned cell to assign pH values to primary standards for pH measurement.

The IUPAC pH scale is valid only for solutions with *I* ≤ 0.1 mol·kg^−1^ according to the Debye–Hückel limiting law applied to electrolytes (at infinite dilution, *γ*_H+_ ≈ 1). This limitation restricts the scale’s applicability to specific conditions [24].

#### Analytical Sensing Using the pH Scale

pH is commonly measured using a pH metre connected to a hydrogen-ion-sensitive glass electrode and a reference electrode, forming an electrochemical cell with a liquid junction (LJ), designated as cell V [44].

RE | KCl [3.5 M] ¦¦ Solution [pH(S) or pH(X)] | GE(Cell V)

RE is the Ag/AgCl reference electrode (filled with 3.5 M KCl or saturated KCl), GE is the glass electrode sensitive to H^+^ and ¦¦ represents a LJ between the reference electrode and the solution being measured, e.g., a standard buffer pH(S) or a sample pH(X).

In cell V, the potential *E* (V) varies linearly with the natural logarithm of hydrogen-ion activity, as described by the Nernst Equation:

(12) $$E=E^{0^{\prime }}+\frac{\mathrm{RT}}{F}\ln\left(a_{H^{+}}\right),$$

where the first term in the right-hand side of Equation (12) is given by:

(13) $$E^{0^{\prime }}=E^{0}+\frac{\mathrm{RT}}{F}\ln{\left(a_{{\mathrm{Cl}}^{-}}\right)}_{\mathrm{KCl}}+E_{j}.$$

In Equation (13), *E*^0^ (V) is the standard potential of the cell and *E*j (V) is the liquid-junction potential (LJP) between the two half cells [24,44]. The relationship between pH(X) and pH(S) is given by Equation (14).

(14) pH(X)= pHS+ES−EX FR T ln 10.

The primary source of measurement uncertainty in pH values obtained using glass electrodes is the LJP between the standard buffer solutions and the sample. This uncertainty can be minimized by ensuring that the *I* of the buffer and the sample are similar, such that the difference in LJP (Δ*E*_j_) between measurements made in the sample (X) and the standard buffer (S) approaches zero (Δ*E*_j_ ≈ 0 V) [48].

In this context, pH measurements are traceable to the IUPAC activity scale if the pH value of the standard has been conventionally assigned by Harned cell measurements, pH(S) = −lg *a*_H_ (S) [49].

Seawater is a complex medium with a high ionic strength (*I* ≥ 0.7 mol·kg^−1^), while conventional pH measurements using glass electrodes and IUPAC buffers (*I* ≤ 0.1 mol·kg^−1^) can introduce a Δ*E*_j_ that is difficult to quantify [32]. The magnitude of this potential is strongly influenced by the *S*, *T*, and *P* of the seawater sample, as well as the composition of the reference buffer solutions and the design of the electrode junctions. As a result, these measurements introduce errors that exceed the desired uncertainty levels for accurately monitoring ocean acidification [45,50].

This implies that for saline solutions, alternative sensor methodologies are necessary for accurately determining pH values [51,52].

### 2.2. Seawater pH Scales

In oceanographic measurements, pH scales are based on the [H^+^] ions rather than on the IUPAC activity scale, because the high *I* of seawater alters ion activity.

Usually, concentration scales in the field of marine chemistry are reported either as molality (mol·kg^−1^) per unit mass of solvent, as molarity (mol·L^−1^), or as amount content (mol·kg^−1^) per unit mass of solution. Therefore, pH values should be reported with care, clearly specifying the scale and concentration units used [42].

Using concentration scales requires making certain assumptions, such as treating the standard state as a constant ionic medium (*γ*_i_ = 1), which implies that the ionic concentration and activity in seawater are identical. The accepted primary reference buffers for determining pH in seawater are nitrogenous base TRIS buffers (2-amino-2-hydroxymethyl-1,3-propanediol), which are effective for *S* ranging from 20 to 40. These buffers have an *I* similar to that of the seawater matrix and are particularly suitable for potentiometric and spectrophotometric pH calibration, as they minimize the Δ*E*_j_. They are prepared using artificial seawater (ASW) as the solvent with varying molalities of HCl [10,22,23,24,30,50]. The pH values are assigned from potentiometric measurements performed using the Harned cell method. The ASW medium does not contain the natural minor halogen ions, as they can contaminate the Ag/AgCl electrodes [42,53].

Detailed procedures for preparing these solutions are extensively described in the literature [10,22,23,30,32,50,54,55,56] along with guidelines from the International Association for the Physical Sciences of the Oceans (IAPSO, 2008) [57,58].

#### 2.2.1. The pH_F_ Scale

The free proton pH scale, pH_F_, focuses on the “free” fraction of H^+^ ions that is not bound to other chemical species in seawater, as defined in Equation (15).

(15) pHF=−lg H+b0,

where [H^+^] is the molal concentration of free hydrogen ion H^+^ in mol·kg^−1^ of seawater solution and *b*^0^ is the standard molality (1 mol·kg^−1^). It is worth noting that the pH_F_ scale is similar to the pH scale proposed by Søren Sørensen in 1909, which was defined in terms of concentration rather than activity; the Debye–Hückel theory of ionic interactions was introduced later [42,48,59]. The pH_F_ of reference buffer solutions is assigned by Harned cell measurements in the cell configuration (A) [49,60].

Pt | H_2_ (g, 1 atm) | ASW with HA and HB | AgCl |Ag, (A)

where HA and HB are the acidic and basic forms of the added buffer solution, respectively [53]. The *E* (V) measurements in the cell (A) are related to pH according to Equation (16).

(16) pHF=E−Eb0FRTln10+lgbClb0+2 lgγHCl±γtrHCl±.

where *E* (V) is the potential of the electrochemical cell with buffer solution in an ASW medium, *E*^0^*_b_* (V) is the standard potential of the Ag/AgCl electrodes measured in ASW with HCl added, *F* is the Faraday constant (96,485.33212 C·mol^−1^), *R* is the molar gas constant (8.3144626 J mol^−1^·K^−1^), *T* is the absolute temperature (K), *b*_Cl_ is the molality of the Cl^−^ ions in the solution (mol·kg^−1^), *b*^0^ is the standard molality (mol·kg^−1^), $\gamma_{\mathrm{HCl}}^{\pm }$ is the average activity coefficient of HCl and ${\left(\gamma^{\mathrm{tr}}\right)}_{\mathrm{HCl}}^{\pm }$ is the trace activity coefficient of the medium [53]. The quotient in the third term on the right-hand side of Equation (16) is 1 when [HCl] = 0 [61]. *E*^0^*_b_* is measured experimentally with the Harned cell using the cell configuration (B).

Pt | H_2_ (g, 1 atm) | ASW with HCl | AgCl | Ag (B)

The determination of *E*^0^*_b_* is made according to Equation (17).

(17) Eb0=limbHCl→0E(ASW+HCl)+RT ln(10)FlgbHbCl,

where *E_b_*^0^ is a limit, the intercept of a second-order polynomial of *E_(_*_ASW+HCl)_ as a function of *b*_HCl_ at zero molality [30,52,59,60].

A limitation of this scale is that, in natural seawater, some H^+^ ions are chemically bound to sulfate ions (SO_4_^2−^). Therefore, [H^+^] is calculated using Equation (18).

(18) $$\left[H^{+}\right]=\frac{\left[\mathrm{HCl}\right]}{1+\left(\frac{\left[{\mathrm{SO}}_{4}^{2-}\right]}{K_{{\mathrm{HSO}}_{4}}^{*}}\right)}$$

In seawater, *K*_HSO4_^−^ (the ionization constant of the anion HSO_4_^−^) and $\gamma_{\mathrm{HCl}}^{\mathrm{tr}}$ are not known [53]. In the work of Khoo et al. [60], the trace activity of HCl was calculated using ASW buffered solutions prepared without the SO_4_^2−^ ion, which was replaced with NaCl to maintain the formal *I*. The trace activity coefficient $\left(\gamma_{\mathrm{HCl}}^{\mathrm{tr}}\right)$ was then estimated as a reasonable approximation for solutions containing SO_4_^2−^, such as seawater. Later, in the work of Bates and Erickson [62], buffered ASW solutions containing sulfate were prepared, and the trace activity of HCl was individually estimated in simplified electrolyte solutions containing the most prevalent salts in seawater, such as NaCl, KCl, and others.

Mathematical models such as the Pitzer formalism (an extension of the Debye–Hückel law to *I* > 0.1 mol·kg^−1^) are used to estimate the activity coefficients of HCl in saline media [25,26]. Computer programmes such as MIAMI or FREZCHEM also use data from the CO_2_ system variables to predict pH. However, there is still no consensus among scientists regarding the values obtained at temperatures other than 25 °C or the contribution of uncertainty to pH measurements [42].

The pH_F_ scale is simpler than the other pH concentration scales, such as pH_T_ and pH_SWS_ scales, because it excludes the H^+^ associated with sulfate and other species in seawater, making it useful for the studying of important acid–base equilibria in solution [53].

#### 2.2.2. The pH_T_ Scale

The total proton pH scale (pH_T_) is based on the free [H^+^] and the [H^+^] bound to the SO_4_^2^. This scale was originally proposed by DelValls and Dickson [56] and can be represented by Equation (19) [22,23,24,30,32,46,50,54,56].

(19) pHT =−lg H++HSO4−b0.

The pH_T_ scale is related to the pH_F_ scale by Equation (20) [30].

(20) $$pH_{T}={\mathrm{pH}}_{F}-\mathrm{lg}\left(\frac{1+\left[{\mathrm{SO}}_{4}^{2-}\right]/K_{{\mathrm{HSO}}_{4}}^{*}}{b^{0}}\right),$$

where [SO_4_^2−^] is the total sulfate concentration and $K_{{\mathrm{HSO}}_{4}}^{*}$ is the ionization constant of the ion HSO_4_^−^ expressed in Equation (21).

(21) $$K_{{\mathrm{HSO}}_{4}}^{*}=\underset{\left[H^{+}\right]\to 0}{\lim}\left\{\frac{\left[H^{+}\right]\left[{\mathrm{SO}}_{4}^{2-}\right]}{\left[{\mathrm{HSO}}_{4}^{-}\right]b^{0}}\right\}.$$

As with the pH_F_ scale, the assignment of pH_T_ values to primary buffer solutions is performed operationally using a Harned cell with the configuration (A) [22,23,30,32]:

(22) pHb=E−E0*FRT ln(10)+lgbClb0,

where *E* (V) is the potential difference (Δ*E*) measured between the Pt/H_2_ and the Ag/AgCl electrodes immersed in the TRIS buffer solution, *E*^0*^ (V) is the standard potential of the Ag/AgCl electrodes in an ionic medium such as seawater and needs to be determined, and *b*_Cl_ (mol·kg^−1^) is the total molality of Cl^−^ in the buffer solution.

To determine *E*^0*^, ASW solutions containing HCl at increasing molalities (with *S* and *I* matching the buffered solution) are measured using the Harned cell configuration (B); *E*^0*^ is then calculated by Equation (23).

(23) $$E^{0*}=\underset{b_{\mathrm{HCl}}\to 0}{\lim}E^{\prime }.$$

*E*^0*^ (V) is determined using the statistical least squares regression as the intercept at zero molality of HCl of a second-order polynomial fit of *E*’ as a function of *b*_HCl_ as expressed by Equation (24).

(24) $$E^{\prime }=E_{\left(\mathrm{ASW},\mathrm{HCl}\right)}+\frac{R\;T\ln10}{F}\cdot \mathrm{lg}\left(b_{\mathrm{HCl}}\cdot b_{{\mathrm{Cl}}^{-}}\right)$$

where *E*_(ASW, HCl)_ (V) is the Δ*E* between the electrodes immersed in the ASW solutions with increasing molalities of HCl.

In Equation (22), pH*_b_* is calculated by measuring the Δ*E* (V) between the electrodes immersed in buffer solutions prepared on a molality basis, in mol·kg^−1^ of solvent. pH_T_ of seawater is expressed on an amount-content basis, in mol·kg^−1^ of solution, and is converted using the *S*-dependent correction term as defined in the work of DelValls and Dickson [56] and adopted by several researchers to ensure comparability between results [23,30,46].

pH_T_ is then calculated using Equation (25).

pH_T_ = pH*_b_* − lg(1 − 0.00106 *S*) (25)

The term (1 − *S* 0.00106) in Equation (25) links *S* to the water mass fraction (*ω*_H2O_) as determined for ASW without TRIS addition [30] with the composition and atomic weights available in 1998. This introduces two small inaccuracies: TRIS/TRIS.HCl addition alters the *ω*_H2O_ relative to the unbuffered ASW [54], and salt molalities calculated from *S* have since been refined using updated atomic weights [63,64]. To avoid both, Pratt [54] and Capitaine et al. [22,32] calculate *ω*_H2O_ from the weighed buffer composition, converting pH*_b_* to pH_T_ with Equation (26).

(26) pHT=E−E0*FRT ln(10)+lgbClb0−lg(ωH2O)

Quendera et al. [23] compared pH_T_ values obtained using Equation (25) with published pH_T_ values from Pratt [54] and Capitaine et al. [32], obtained using Equation (26). The two conversions agreed within the combined uncertainties (*E*_n_ < 1).

TRIS buffers characterized using the Harned cell are considered to have the highest metrological quality and rigour and serve as reference for all other pH_T_ measurements in seawater [23,24,46,49].

Recent studies by Clegg et al. [25], Humphreys et al. [26] and Clegg et al. [27] employed chemical-speciation models based on the Pitzer activity coefficient equations to predict the behaviour of TRIS buffers in ASW and in natural seawater. These studies examined the pH_T_ scale under various conditions, including ASW at 25 °C, ASW across a temperature range from 0 °C to 45 °C, and natural seawater from the freezing point to 45 °C. The corresponding acid–base equilibria were also modelled, with a particular focus on accurately accounting for uncertainty propagation.

The pH_T_ of seawater can also be determined using optical methods, e.g., spectrophotometric techniques. These techniques are increasingly used in oceanography because of their relative simplicity, speed and accuracy. The principle of spectrophotometric pH_T_ measurements relies on the fact that adding an indicator (such as meta-cresol purple, cresol red or bromocresol green) to a seawater sample results in a colour change corresponding to variations in the sample’s pH. This colour change is determined by the physicochemical properties of the indicator, which are reflected in its absorbance spectra, as a variation in the absorbance peak ratio (*R*) [28,43,65]. Today, meta-cresol purple is the most widely used and well-characterized indicator dye for spectrophotometric pH_T_ measurements in seawater [22,24,29,42].

The pH_T_ of the solution (seawater and indicator) is expressed by Equation (27).

(27) $${\mathrm{pH}}_{T}={pK}_{\mathrm{ind}}+\mathrm{lg}\left(\frac{\left[I^{2-}\right]}{{[\mathrm{HI}}^{-}]}\right),$$

where the species [I^2−^] and [HI^−^] represent the concentrations of the basic and acidic forms of the indicator, respectively, and p*K*_ind_ is the acid ionization constant of the indicator, defined by Equation (28).

(28) $${pK}_{\mathrm{ind}}=-\mathrm{lg}\left(K_{{\mathrm{HI}}^{-}}\right),$$

Therefore, $K_{{\mathrm{HI}}^{-}}$ is expressed as:

(29) $$K_{{\mathrm{HI}}^{-}}=\frac{\left[I^{2-}\right]\left[H^{+}\right]}{\left[{\mathrm{HI}}^{-}\right]}.$$

Equation (27) can also be rewritten as Equation (30).

(30) $${\mathrm{pH}}_{T}=-\mathrm{lg}\left(K_{{\mathrm{HI}}^{-}}\right)+\mathrm{lg}\left(\frac{R-e_{1}}{e_{2}-R\frac{e_{3}}{e_{2}}}\right),$$

where *R* is the ratio of absorbances *A*_2_ and *A*_1_ at wavelengths *λ*_2_ and *λ*_1_:

(31) $$R=\frac{A_{2}}{A_{1}},$$

for the m-cresol purple indicator, *λ*_2_ = 434 nm and *λ*_1_ = 578 nm. The molar absorptivity coefficients (*e*_1_, *e*_2_ and *e*_3_) are defined as follows by Equation (32):

(32) $$e_{1}=\frac{\varepsilon_{2({\mathrm{HI}}^{-})}}{\varepsilon_{1({\mathrm{HI}}^{-})}};e_{2}=\frac{\varepsilon_{2\left(I^{2-}\right)}}{\varepsilon_{1\left({\mathrm{HI}}^{-}\right)}};e_{3}=\frac{\varepsilon_{1\left(I^{2-}\right)}}{\varepsilon_{1\left({\mathrm{HI}}^{-}\right)}},$$

where *ε*_1_ and *ε*_2_ represent the molar absorptivity of HI^−^ and I^2−^ at wavelengths *λ*_1_ and *λ*_2_, respectively. *K*_HI−_, *ε*_1_ and *ε*_2_ are all dependent on *S* and the *T*.

The studies conducted by Douglas and Byrne [28], Li et al. [29] and Liu et al. [66] characterized the parameters *K*_HI−_, *e*_1_, *e*_2_ and *e*_3_ in natural water, seawater and/or ASW for indicators intended for future spectrophotometric pH_T_ sensor applications. Their work achieved a reproducibility of 0.0004. Other researchers [43,66] have utilized these characterized indicators for spectrophotometric pH_T_ measurements in seawater and ASW, achieving an accuracy of 0.003.

#### 2.2.3. The pH_SWS_ Scale

The seawater pH scale or pH_SWS_ was initially proposed by Hansson [67] for the study of the thermodynamic variables of the CO_2_ system. It incorporates important chemical species participating in the acid–base equilibria in seawater (the bisulfate and fluoride ions), making it conceptually more complete than the pH_F_ and the pH_T_ scales [53]. The pH_SWS_ scale considers both the free H^+^ ions and those chemically bound to SO_4_^2−^ (HSO_4_^−^) and F^−^ (HF) ions [53].

In general, pH_SWS_ can be expressed by Equation (33) and more accurately by Equation (34).

(33) pHSWS ≈−lg bH++bHSO4−+bHFb0,

where $b_{H^{+}}$, $b_{{\mathrm{HSO}}_{4}^{-}}$, and *b*_HF_ are the molalities of H^+^, HSO_4_^−^ and HF, respectively.

(34) $$pH_{\mathrm{SWS}}=-\mathrm{lg}\left(\frac{\left[H^{+}]+[{\mathrm{HSO}}_{4}^{-}]+[\mathrm{HF}\right]}{b^{0}}\right),$$

According to Waters and Millero [53], pH_F_, pH_T_ and pH_SWS_ scales are related (Equations (15), (19), (20) and (34)). Equation (35) establishes the conversion between these scales:

(35) pHSWS =pHF−lg 1+SO42−KHSO4+F−KHFb0,

where *K*_HSO4_ and *K*_HF_ are the dissociation constants of the (HSO_4_^−^) and hydrogen fluoride (HF), respectively, and [SO_4_^2−^] and [F^−^] are the molal concentrations of the bisulfate and fluoride ions (SO_4_^2−^and F^−^), respectively, in mol·kg^−1^.

The pH_SWS_ values are converted to an amount-content basis, in mol·kg^−1^ solution, using Equation (36).

(36) pHSWS =pHF−lg 1+SO42−KHSO4+F−KHFb0−lg 1−1.005×10−3S,

The fluoride ion (F^−^) is present in trace concentrations in seawater, leading to a difference of approximately 0.01 between pH_T_ and pH_SWS_. Due to this small difference and its accuracy, the pH_T_ scale is the most widely used in oceanographic studies [24,52,53,54].

### 2.3. The pH_abs_ Scale

The unified pH scale, pH_abs_, was introduced by Himmel et al. [33] in 2010 on the basis of proton solvation free energies, enabling comparison of strong-acid acidity across different solvents. Its analytical and metrological realization for aqueous and non-aqueous media was developed through the EMPIR project UnipHied (2018–2021) [34,35,40,68]. An important feature of the theoretical pH_abs_ concept is that the zero point is common to all media, allowing acidity to be compared across aqueous and non-aqueous phases [33,69]. This thermodynamic concept must be distinguished from its current experimental realization. Present realizations use differential potentiometry, glass electrodes, conventional anchor buffers, and an ionic liquid salt bridge. The limitations caused by residual junction potentials and the uncertainties discussed in Section 2.3.1 and Section 2.3.2 therefore apply to this realization and should not be interpreted as an intrinsic failure of the chemical-potential definition. The scale is defined in terms of the absolute standard chemical potential of the proton, *μ*^0^_abs_(H^+^), where the reference is the absolute chemical potential of the proton, *m*_abs_(H^+^) in the gas phase (one bar, 298.15 K) which is arbitrarily defined as 0 kJ·mol^−1^. The unified pH scale expresses proton chemical potential on a common reference scale. Within a specified medium, proton chemical potential is related to proton activity through the corresponding standard-state chemical potential. Thus, pH_abs_ should not be interpreted as a hydrogen-ion concentration or assumed to be numerically interchangeable with operational seawater pH scales without specifying the reference state and the relationship between the scales.

The chemical potential *μ*(H^+^) in the gas phase or in solution can generally be described by a standard chemical potential *μ*^0^(H^+^) and a concentration-dependent activity term as given by Equation (37).

(37) μH+=μ0H++RTln aH+=μ0H+−RT ln10×pH.

In Equation (37), *μ*(H^+^) is a linear quantity that expresses energy (kJ·mol^−1^) whereas pH is, by definition, a logarithmic function of *a*_H+_ (pH = −lg *a*_H+_). The two are rigorously and linearly related because the logarithm is already embedded in the standard thermodynamic definition of the chemical potential of a solute, *μ* = *μ*^0^ + *RT* ln *a*. No additional logarithmic transformation is introduced when moving from *μ* to pH [44,70].

The absolute chemical potential of the proton, *μ*_abs_ (H^+^), is defined as:

(38) $$\mu_{\mathrm{abs}}\left(H^{+}\right)=\mu \left(H^{+}\right)-\mu_{\mathrm{abs}}^{0}\left(H^{+}\right),$$

where *μ*^0^_abs_(H^+^) is the absolute standard chemical potential of H^+^. When *μ*^0^_abs_(H^+^) = 0 kJ·mol^−1^ (by definition), the standard Gibbs free energy of solvation, Δ_solv_*G*^0^(H^+^), can be used to construct a pH_abs_ scale in a given solvent as expressed by Equation (39).

*μ*_abs_(H^+^, solv) = ∆_solv_*G*^0^(H^+^)−pH × 5.71 kJ·mol^−1^, (39)

where *R* is the molar gas constant (8.3144626 J mol^−1^·K^−1^), *T* is the absolute temperature in K, pH is the conventional (solvent-specific) IUPAC pH scale and *μ*_abs_ (H^+^, solv) is the absolute chemical potential of the solvated proton [33,35]. For a change of one pH unit (ΔpH = 1) at 298.15 K, Himmel et al. [33] show that:

(40) $$\left|∆\mu_{\mathrm{abs}}\left(H^{+}\right)\right|=\mathrm{RT}\left|∆\ln\left(a_{H^{+}}\right)\right|=2.303\mathrm{RT}\left|∆\mathrm{lg}\left(a_{H^{+}}\right)\right|=2.303\mathrm{RT}=5.71\mathrm{kJ}\cdot {\mathrm{mol}}^{-1},$$

Thus, one pH unit is equivalent to a chemical potential difference of 5.71 kJ·mol^−1^ at 298.15 K and pH_abs_ is therefore defined by Equation (41).

(41) $${\mathrm{pH}}_{\mathrm{abs}}=-\frac{\mu_{\mathrm{abs}}\left(H^{+},\mathrm{solv}\right)}{5.71\mathrm{kJ}/\mathrm{mol}}.$$

Combining Equations (39) and (41) at pH = 0, in water, gives *μ*_abs_(H^+^, H_2_O) = Δ_solv_G^0^(H^+^, H_2_O) = −1105 kJ mol^−1^ [33,35].

Therefore, pH_abs_(H_2_O, pH = 0) = −(−1105/5.71) ≈ 193.5. In this context, pH_abs_ values are shifted by a constant (−193.5 pH units, at 298.15 K), allowing this scale to be directly comparable with conventional IUPAC pH values or ${\mathrm{pH}}_{\mathrm{abs}}^{H_{2}O}$ [35].

This derivation exposes a more consequential point obtained directly from Equations (39) and (41), giving the general relation between ${\mathrm{pH}}_{\mathrm{abs}}^{H_{2}O}$ and the conventional pH scale in a solvent (S) anchored to Δ_solv_*G*^0^(H^+^, H_2_O) as expressed by Equation (42).

(42) pHabsH2O=−ΔsolvG0H+, SRT ln10+pHS+ΔsolvG0H+,H2ORT ln10.

Equation (42) can be simplified using the same relation in terms of the Gibbs energy of transfer between water and S, Δ_tr_*G*^0^(H^+^, H_2_O → S), expressed by Equation (43).

∆_tr_*G*^0^(H^+^,H_2_O → S) = ∆_solv_*G*^0^(H^+^,S) − ∆_solv_*G*^0^(H^+^,H_2_O). (43)

Substituting Equation (43) into Equation (42) to eliminate Δ_solv_*G*^0^(H^+^, S), the two terms in Δ_solv_*G*^0^(H^+^, H_2_O) cancel out giving Equation (44).

(44) pHabsH2O=−ΔtrG0H+,H2O →SR T ln10+pHS

In Equation (44) it is explicit that ${\mathrm{pH}}_{\mathrm{abs}}^{H_{2}O}$ is anchored to water: setting S = H_2_O gives Δ_tr_*G*^0^(H^+^, H_2_O → H_2_O) = 0 (by definition), so Equation (44) reduces to ${\mathrm{pH}}_{\mathrm{abs}}^{H_{2}O}$ = pH_H2O_, which is precisely the conventional IUPAC pH scale. However, this holds only for water as the reference solvent where the conventional IUPAC pH scale is valid (*I* ≤ 0.1 mol·kg^−1^) [44]. For seawater, it additionally requires that Δ_solv_*G*^0^(H^+^, seawater) ≈ Δ_solv_*G*^0^(H^+^, H_2_O), i.e., that Δ_solv_*G*^0^(H^+^) is not altered by the high *I* and ionic composition of seawater relative to dilute water. This assumption is neither derived nor tested within the pH_abs_ scale definition, and its validity is precisely what the studies in seawater test experimentally as discussed further in Section 2.3.2.

For a fixed aqueous medium, temperature, pressure, and consistent standard-state convention, ${\mathrm{pH}}_{\mathrm{abs}}^{H_{2}O}$ and an activity-based pH description are related through the proton chemical potential. This relationship does not, by itself, establish numerical equivalence with the operational free, total, or seawater pH scales. In seawater, comparisons require explicit consideration of medium composition, activity conventions, and the calibration and conversion procedures used.

A principal motivation for the unified pH framework is the comparison of proton chemical potentials across different media. Its application to seawater may therefore be conceptually useful, particularly when comparing media of different compositions. However, the experimental studies reviewed here do not yet demonstrate a metrological advantage over established seawater pH procedures. This assessment concerns the reported implementations and their uncertainty budgets, rather than an intrinsic limitation of the thermodynamic framework. In this context, the ${\mathrm{pH}}_{\mathrm{abs}}^{H_{2}O}$ concept is intended for the quantitative comparison of acidity across different solvents (e.g., water, acetonitrile, DMSO, or aqueous and non-aqueous solvents) [33,34,36,69,71], a comparison that solvent-specific pH scales cannot make.

Neither ${\mathrm{pH}}_{\mathrm{abs}}^{H_{2}O}$ nor an operational seawater pH value uniquely determines CO_2_ fugacity. At gas–liquid equilibrium, CO_2_ fugacity is related to dissolved CO_2_ through the solubility coefficient, whereas proton chemical potential is linked to carbonate speciation through acid-base equilibria. The relationship therefore depends on temperature, salinity, pressure, and additional carbonate-system information, such as total alkalinity. Under otherwise comparable conditions and approximately constant total alkalinity, increasing CO_2_ fugacity generally lowers seawater pH; this conditional relationship is not a definition of pH_abs_.

#### 2.3.1. Differential Potentiometry as the Reference Method for pH_abs_ Measurement

Differential potentiometry was selected as the reference method for the analytical realization of ${\mathrm{pH}}_{\mathrm{abs}}^{H_{2}O}$ within the European project EMPIR 17FUN09-UnipHied (2018–2021) [40,68]. The investigated matrices were non-aqueous solvents, namely acetonitrile, ethanol and methanol and their mixtures with water.

Unlike conventional potentiometric sensors (Section Analytical Sensing Using the pH Scale), which require calibration buffers of matched *I* and ionic composition to minimize the Δ*E*_J_ between standards and samples, the ${\mathrm{pH}}_{\mathrm{abs}}^{H_{2}O}$ method is intended to be independent of solvent and instrumentation; it relies on a nearly ideal salt bridge, rather than on matrix matching, to suppress the LJP regardless of the media on either side of the cell [35]. The ${\mathrm{pH}}_{\mathrm{abs}}^{H_{2}O}$ was measured experimentally using two glass electrodes sensitive to the H^+^ ion, immersed in two solutions with different compositions, each contained in one of two symmetrical glass cells. The cells were connected to an instrument capable of measuring the potential difference between the electrodes, such as a pH meter, multimeter, electrometer, or potentiostat. This method serves as the reference technique for obtaining ${\mathrm{pH}}_{\mathrm{abs}}^{H_{2}O}$ values in solutions [34,35,36,37,39,71].

**Figure 3:**
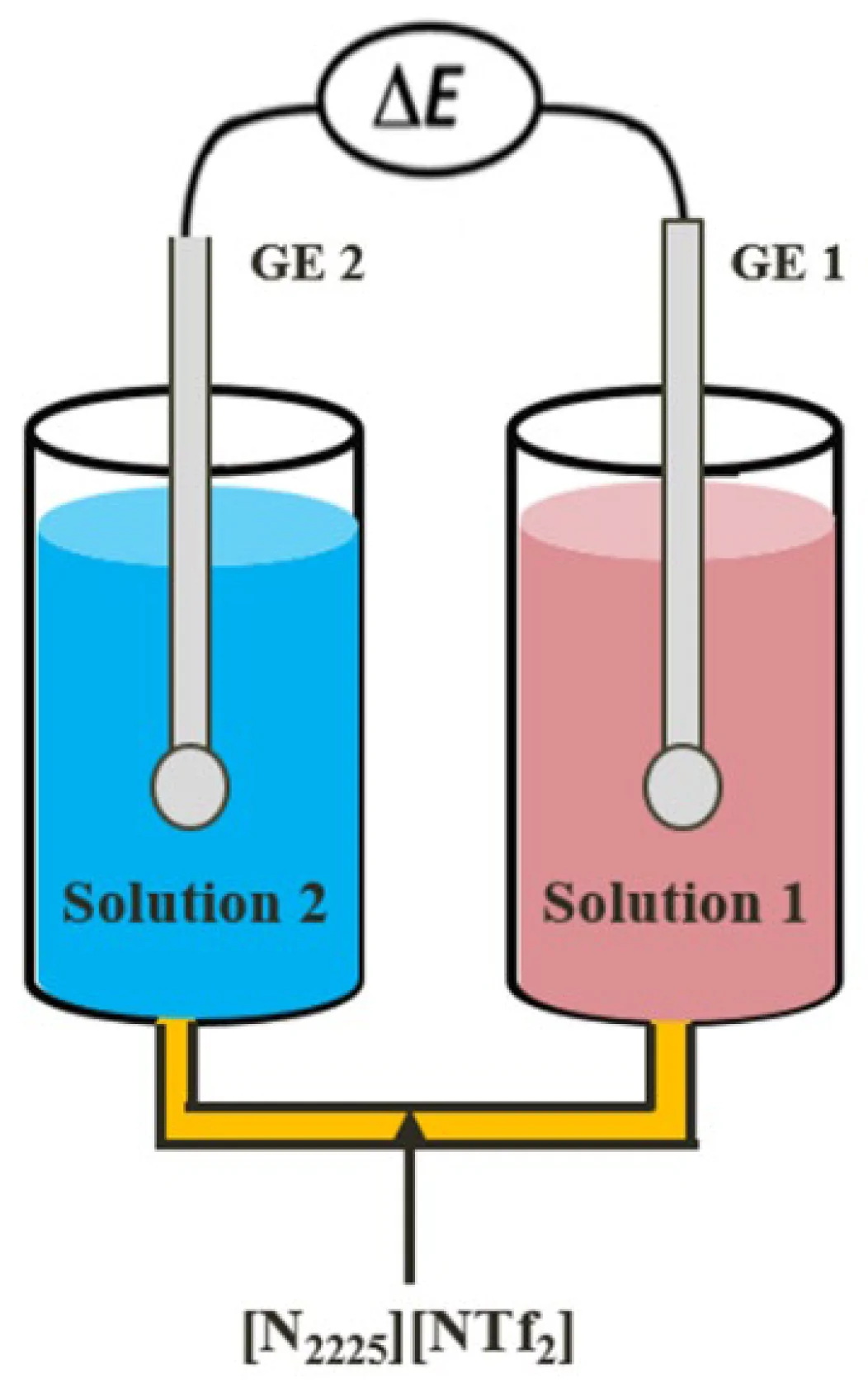
Schematic representation of a potentiometric cell for the measurement of reference ${\mathrm{pH}}_{\mathrm{abs}}^{H_{2}O}$ values by differential potentiometry (adapted from [34]).

The symmetrical glass cells are separated by a salt bridge containing the ionic liquid [N2225][NTf2] (triethylpentylammonium bis(trifluoromethanesulfonyl)imide), allowing direct measurement of the potential between the electrodes. The ionic liquid was selected because, as a strong binary electrolyte with cations and anions of similar transference numbers and no solvent of its own, it approximates an ideal salt bridge [35,38]. In practice, the two junction potentials formed at the ends of the capillary do not cancel perfectly. The IUPAC technical report [35] reports a residual liquid-junction potential (RLJP) of 0 ± 6.3 mV, obtained from a statistical analysis of the consistency of 87 cell combinations. In the UnipHied project, 6.3 mV was subsequently adopted as a standard uncertainty (*k* = 1) [37,72]. Here, this value is used to illustrate the potential contribution of RLJP to pH measurement uncertainty. At 298.15 K, the ideal Nernst sensitivity is approximately 59.16 mV per pH unit, yielding a standard uncertainty of *u*_RLJP_(pH) ≈ 0.1 and an expanded uncertainty of *U*_RLJP_(pH) ≈ 0.2 for *k* = 2. These values quantify only the potential RLJP contribution; they do not constitute a complete measurement uncertainty budget. The estimate of 6.3 mV [35] was obtained for dilute solutions and has not been validated as an uncertainty contribution due to the residual liquid-junction potential (RLJP) in seawater. Its conversion into pH units is therefore illustrative and does not establish the corresponding uncertainty for seawater or brines. The results reported for saline matrices [37,39] indicate limitations of the investigated ionic liquid salt bridge under the conditions studied; they do not establish a single uncertainty applicable to all seawater measurements. Extrapolation to other seawater samples or brines requires experimental validation under the relevant measurement conditions. The schematic representation of a symmetric cell used for differential potentiometry is shown in Figure 3.

The following configuration represents the electrochemical cell in Figure 3.

GE2 | S2 | [N_2225_][NTf_2_] | S1 | GE1, (C)

where GE1 and GE2 are the glass electrodes sensitive to H^+^, and S1 and S2 represent the solutions under study.

The system is calibrated with commercial pH buffer solutions traceable to the IUPAC pH scale. The slopes and intercepts of both electrodes (GE1 and GE2) are obtained by measuring the potential *E* (mV) of the glass electrodes against a commercial Ag/AgCl reference electrode.

The potential difference Δ*E* (mV) measured between the two glass electrodes (GE1 and GE2) is converted into Δ${\mathrm{pH}}_{\mathrm{abs}}^{H_{2}O}$ according to:

(45) $$\Delta {\mathrm{pH}}_{\mathrm{abs}}^{H_{2}O}=\frac{\Delta E-I_{\mathrm{mean}}}{s_{\mathrm{mean}}}$$

where *I*_mean_ and *s*_mean_ are the mean intercept and mean slope of the glass electrodes, respectively, obtained after calibration. The assignment of ${\mathrm{pH}}_{\mathrm{abs}}^{H_{2}O}$ values to the solutions/samples under study is carried out by measuring $\Delta {\mathrm{pH}}_{\mathrm{abs}}^{H_{2}O}$ in different combinations of solutions in the symmetrical glass cells, using an approach called the “ladder” method. The ${\mathrm{pH}}_{\mathrm{abs}}^{H_{2}O}$ values are assigned by applying a nonlinear least squares regression method to the measured $\Delta {\mathrm{pH}}_{\mathrm{abs}}^{H_{2}O}$ values, using buffer solutions of known pH as anchor points [34,35,36,37,39,71]. These anchor points are conventional IUPAC traceable buffers; anchoring this scale to IUPAC buffers is how the scale is linked to a known reference and is conceptually distinct from the matrix-matching requirement that limits conventional glass electrode sensors in saline media. Therefore, the $\Delta {\mathrm{pH}}_{\mathrm{abs}}^{H_{2}O}$ measurement does not require that anchor buffers share the same *I* or matrix of the samples, because that role is performed by the ionic liquid salt bridge (ILSB).

The practical limitation lies in how effectively the ionic liquid salt bridge cancels the two junction potentials, which depends on *I*. As shown above, the corresponding standard uncertainty is *u*_pH_ ≈ 0.1 at 25 °C, yielding *U*_pH_ ≈ 0.2 for *k* = 2. Lainela et al. [37] demonstrate that the [N_2225_][NTf_2_] ILSB deteriorates as *I* increases. Because the reported ±6.3 mV standard uncertainty was established for dilute solutions, it cannot be considered as directly representative of seawater conditions. The main obstacle is not the re-emergence of the conventional matrix-dependent calibration problem but the growing contribution of RLJP at elevated *I*. As a result, the achievable accuracy of ${\mathrm{pH}}_{\mathrm{abs}}^{H_{2}O}$ measurements in saline media is ultimately constrained by the incomplete cancellation of junction potentials across the ILSB.

The reference method and a potentiometric approach were investigated, employing commercial glass electrodes coupled with a double-junction reference electrode that contained an IL in the outer compartment. The robustness of the ${\mathrm{pH}}_{\mathrm{abs}}^{H_{2}O}$ in non-aqueous matrices and their mixtures with water was demonstrated through an interlaboratory comparison [71].

#### 2.3.2. The ${\mathrm{pH}}_{\mathrm{abs}}^{H_{2}O}$ Scale Applied to Saline Solutions: Critical Assessment

The studies reviewed here have not yet demonstrated a metrological or practical advantage of the current differential-potentiometric realization of ${\mathrm{pH}}_{\mathrm{abs}}^{H_{2}O}$ over established operational seawater pH procedures.

Lainela et al. [37] conducted experiments in simulated estuarine systems. Primary pH values of TRIS-TRIS.HCl buffer solutions in ASW (composed of NaCl only, *S* = 20) were assigned through Harned cell measurements, and the Pitzer formalism was used to assign *γ*_Cl_^−^. Two approaches were used to assess ${\mathrm{pH}}_{\mathrm{abs}}^{H_{2}O}$ in saline solutions. The first approach was based on conventional potentiometry with a pH metre and glass electrodes, using IUPAC buffer solutions to calibrate the system and measuring the pH of the TRIS-TRIS.HCl buffer solutions as unknown samples, reproducing current routine practice for seawater pH measurements. The second approach was based on the ${\mathrm{pH}}_{\mathrm{abs}}^{H_{2}O}$ scale with differential potentiometry, using the reference method and a simpler potentiometric method with commercial glass electrodes and a double-junction reference electrode. Separately, the RLJP associated with both KCl and ILSBs was evaluated directly using NaCl solutions with 0.005 ≤ *I* ≤ 0.8 mol·kg^−1^, spanning dilute solutions up to values close to the *I* of seawater (*I* ≈ 0.7 mol·kg^−1^). For *I* ≤ 0.1 mol·kg^−1^, the activity coefficients were calculated using the Debye–Hückel equation, while for the NaCl solutions with higher *I*, the Pitzer equations were applied. The LJP were more effectively minimized when electrodes with a KCl bridge were used, than when electrodes with an ILSB were used. Lainela et al. [37] showed that RLJP increase with *I* and become larger for the [N_2225_][NTf_2_] ILSB than for a KCl salt bridge. Therefore, in the saline media investigated, the conventional KCl bridge yielded more accurate pH measurements. The ${\mathrm{pH}}_{\mathrm{abs}}^{H_{2}O}$ values obtained for the TRIS-TRIS.HCl buffers also closely resembled the pH values obtained by conventional calibration with IUPAC buffers.

In the work of Quendera et al. [39], saline solutions were prepared from standard seawater supplied by Ocean Scientific International Laboratory (OSIL), with absolute salinity *S*_A_ = 37 g·kg^−1^ [73,74]. The standard seawater was adjusted with NaCl to values up to *S*_A_ = 230 g·kg^−1^, approaching brine salinity, and compared with ultrapure water/NaCl solutions covering 20 ≤ *S*_A_ ≤ 230 g·kg^−1^. In the pure NaCl solutions, the difference between pH_abs_ and conventional potentiometric pH showed no consistent trend with *S* or *I*. In the OSIL solutions, the difference converged toward that observed for pure water. This behaviour was attributed to RLJP effects associated with the current differential-potentiometric realization and calibration with dilute anchor buffers.

The expanded uncertainty reported for pH measurements on the unified pH scale $U_{{\mathrm{pH}}_{\mathrm{abs}}^{H2O}}$= 0.2 [37,39] was consistent with the finding outlined in Section 2.3.1. The major contribution to this uncertainty was the RLJP formed at the ends of the ILSB, which grows as the *I* of the media increases, rather than remaining constant.

In its current experimental realization, the unified pH scale therefore does not provide seawater measurements with the solvent-independent advantage that motivates its theoretical design. Lainela et al. [37] explicitly identify the development of a more suitable IL or combination of ILs as a potential improvement.

Taken together, these findings place the current experimental realization of pH_abs_ in an unfavourable position relative to established seawater pH methods for sensor and monitoring applications. *U*_pH_ ≈ 0.2 (*k* = 2) is approximately 33 times an expanded climate-quality target of *U* ≤ 0.006 (*k* = 2), five times the weather-quality value of *U ≤* 0.04 (*k* = 2). These ratios are presented separately to avoid mixing standard and expanded uncertainties [20,75,76].

## 3. Comparison of pH Scales for Seawater Sensor Applications

The choice of pH scale has direct implications for calibration, traceability, comparability, and measurement uncertainty in oceanographic sensing. Table 1 summarizes the conceptual basis, measurement methodology, representative seawater pH values for the five pH scales, discussed above, for *S* = 35 and *T* = 25 °C, monitoring suitability, and principal references for the scales considered in this review. The numerical values were not obtained from a single seawater sample in a common intercomparison and are included only to illustrate the scale-dependent values reported or calculated under the cited conditions. Differences may therefore reflect both the scale definition and the sample composition, temperature, salinity, and measurement procedure. The ${\mathrm{pH}}_{\mathrm{abs}}^{H_{2}O}$ value is from an OSIL standard-seawater measurement [39]. 

The pH scale must be considered separately from the sensor transduction principle. Potentiometric glass electrodes may report values on the IUPAC, free, total, or seawater scales, depending on the calibration and conversion procedure. They remain widely used because they are inexpensive, familiar, and provide rapid measurements, but seawater measurements are affected by calibration transfer, RLJP, reference-electrode stability, temperature, salinity mismatch, electrode ageing, and drift [10,44,45,47,48,49,55,56,77,78,79,80]. Calibration or quality control with TRIS-TRIS.HCl buffers prepared in artificial seawater improves matrix matching and traceability to marine operational scales [22,23,24,25,26,27,47,48,49,56,77,79].

Anes et al. [77] evaluated the uncertainty of a procedure using a combination glass electrode and high-ionic-strength TRIS buffers, demonstrating that the uncertainty must be attributed to the complete calibration and measurement procedure rather than to the electrode alone.

ISFET-based sensors, including DuraFET instruments, replace the fragile glass membrane with an ion-sensitive field-effect transistor and are attractive for high-frequency autonomous observations because of their compact size, rapid response, and low-power requirements [81,82]. Their performance nevertheless depends on a stable reference system, sensor conditioning, temperature and salinity compensation, calibration coefficients, pressure response, drift, and biofouling. McLaughlin et al. reported that calibrated ISFET instruments can achieve an accuracy of approximately 0.01 pH units or better and stability of about ±0.005 over weeks to months; however, multi-site field comparisons also showed larger offsets when calibration or operational problems occurred [81]. These values describe accuracy, stability, or agreement with discrete measurements and must not be presented as expanded measurement uncertainty unless a complete uncertainty budget is available.

Solid-state metal-oxide electrodes, particularly Ir/IrOx devices, offer mechanical robustness, rapid response, and opportunities for miniaturization [83,84]. Their response is influenced by oxide preparation, surface hydration, redox state, temperature, ionic strength, hysteresis, and long-term drift. Chen and Compton reported a sensitivity of 70.1 ± 1.4 mV pH−1 for an in situ modified iridium electrode intended for seawater measurements [83]. This value indicates a super-Nernstian response and characterizes the pH sensitivity of the Ir(III/IV) peak potential, as reflected in its calibration slope and its variability; it does not represent an uncertainty expressed in pH units. Quantitative comparison with other platforms requires propagation of the calibration-model, potential-measurement, reference-electrode, temperature, drift, and matrix contributions.

Spectrophotometric methods determine pH_T_ from the absorbance ratio of the acidic and basic forms of an indicator, most commonly meta-cresol purple [20,28,29,43,65,66]. They avoid the liquid junction and therefore have a different uncertainty structure from potentiometric sensors. Their performance depends on indicator purity and characterization, dissociation constants, molar-absorptivity ratios, absorbance measurement, temperature and salinity models, blank correction, mixing, optical stability, and the perturbation caused by indicator addition. Under controlled conditions, purified and characterized meta-cresol purple supports performance of approximately 0.003–0.005 pH units, although the reported quantity may be accuracy, intercomparison agreement, or practical uncertainty rather than a harmonized expanded uncertainty [20,28,29,43,65,66,85]. Autonomous spectrophotometric instruments extend this approach to high-frequency field measurements but add contributions from reagent delivery, pump repeatability, tubing, bubbles, reagent ageing, optical-window fouling, power consumption, and biofouling [20,78].

Optical sensor spots and optodes use immobilized indicators whose absorbance or fluorescence changes with protonation state. They can be compact, electrically isolated, and require little or no liquid reagent, but their performance is affected by dye immobilization, leaching, photobleaching, membrane ageing, temperature and salinity cross-sensitivity, response time, hysteresis, optical drift, and fouling [86,87]. Clarke et al. reported a precision of approximately 0.0074 pH units for an immobilized sensor spot [86], whereas Staudinger et al. observed average absolute deviations of approximately 0.019–0.043 in different field deployments [87]. Because these values represent precision or deviation from reference measurements rather than complete uncertainty budgets, they are not directly comparable with the uncertainty estimates reported for some potentiometric or spectrophotometric procedures.

Digital image colorimetry estimates pH from images of an indicator solution, sensor spot, or test strip acquired with a camera or smartphone. It provides an inexpensive option for screening and distributed observations, but quantitative performance depends on illumination, camera spectral response, automatic exposure and white balance, imaging geometry, image compression, optical path length, turbidity, sample colour, and differences among devices [88,89]. Controlled illumination, colour references, device-specific calibration, and multivariate or machine-learning models can reduce these effects, but model validity remains limited to the calibration domain. Artificial intelligence may support image segmentation, multivariate calibration, anomaly detection, and drift correction, but it cannot independently establish metrological traceability or replace validation against reference values over the relevant ranges of salinity, temperature, turbidity, and sample colour [88].

Microfluidic and lab-on-chip systems integrate sample transport, reagent addition, mixing, and optical or electrochemical detection in compact devices with reduced reagent consumption [20,78,90]. Microfluidics is an implementation architecture rather than a separate pH scale or transduction principle; its analytical performance depends on both the selected detector and the fluidic design. Additional contributions include channel geometry, sample and reagent volumes, mixing efficiency, residence time, flow stability, pump or valve repeatability, adsorption, bubbles, optical alignment, material ageing, and biofouling. Performance and uncertainty should therefore be evaluated for each implementation rather than assigned universally to all lab-on-chip sensors [20,78,90,91].

Table 2 is consequently restricted to potentiometric and spectrophotometric platforms, for which seawater calibration procedures, reference systems, quantitative performance, and principal uncertainty contributions are comparatively well documented [10,20,28,29,43,44,45,47,48,49,55,56,65,66,77,78,79,80,90]. The numerical entries retain the terminology used in the source studies. Accuracy, precision, stability, agreement, and measurement uncertainty are identified separately because they are not metrologically interchangeable [92,93].

## 4. Metrological Challenges and Limitation of Operational pH Scales

Seawater pH is reported on several operational scales and is commonly measured using potentiometric or spectrophotometric methods. Both approaches can provide precise results, but their accuracy and metrological traceability depend on the calibration hierarchy, measurement model, sample composition, and experimental conditions [24,28,42]. It is therefore essential to distinguish limitations associated with the definition of a pH scale from those arising from its analytical realization.

Conventional potentiometric measurements frequently employ glass electrodes calibrated with reference buffers traceable to the IUPAC pH scale [44,47]. However, the activity-coefficient convention used to assign conventional primary pH values is restricted to solutions with ionic strength *I* ≤ 0.1 mol·kg^−1^ [44,47]. Seawater, with an ionic strength of approximately 0.7 mol·kg^−1^ at typical oceanic salinity, falls outside this range. Transferring calibration from dilute buffers to seawater can introduce systematic bias through differences in LJP, alongside contributions from electrode response, reference-electrode instability, and drift. Traceability of the calibration buffers alone therefore does not establish the accuracy of the seawater measurement [24,42,45,50,58].

Matrix-matched calibration using TRIS–TRIS·HCl buffers prepared in artificial seawater provides a more appropriate reference for potentiometric measurements. Junction-free Harned-cell measurements support the assignment of reference values on the specified seawater pH scale and the establishment of an associated calibration hierarchy [24,42,45,50,58]. These assignments require specialized facilities, such as those available at national metrology institutes and selected research laboratories [22,24,31,32,42]. Nevertheless, matching ionic strength alone does not guarantee equivalent junction behaviour: differences in ionic composition, salinity, and temperature between the buffer and natural seawater can leave residual junction effects.

For potentiometric measurements, these effects can be reduced through calibration at the relevant salinity and temperature, appropriate selection of bridge electrolytes and junction configurations, reproducible junction geometry, adequate electrode conditioning, and control of measurement conditions. Their residual contribution should be evaluated through calibration checks, bracketing measurements, and comparison with independently established seawater reference values. Any model-based correction requires validation for the relevant sample matrix and inclusion of its uncertainty in the measurement budget. During field deployment, electrolyte depletion, junction clogging, biofouling, and reference-electrode drift further emphasize the need for regular inspection, calibration verification, and documented quality control.

Spectrophotometric measurements on the total hydrogen-ion concentration scale, pH_T_, are increasingly used because of their precision and suitability for automated operation [28]. These measurements contain no liquid junction and consequently have a different uncertainty structure from potentiometric systems that employ one. Principal contributions include indicator purity and characterization, the adopted dissociation and absorptivity models, absorbance-ratio measurement, temperature and salinity, and changes in sample pH caused by indicator addition [29,66].

Indicator impurities can introduce a substantial, pH-dependent bias. Liu et al. [66] reported differences between measurements using unpurified and purified meta-cresol purple of up to 0.010 pH units at pH 7.4 and 0.018 pH units at pH 8.2. Indicator purification and characterization are therefore essential for accurate spectrophotometric measurements. Because indicator addition also changes the sample acid–base state, the quantity added should be minimized and the remaining perturbation assessed through multiple additions or a validated correction model [29].

Long-term autonomous measurements require additional consideration of reagent stability, delivery, and optical performance. Changes in reagent composition or delivered volume, optical-window fouling, light-source and detector drift, bubbles, turbidity, and imperfect temperature control can introduce systematic errors that are not apparent from measurement repeatability alone. Suitable quality-control procedures include baseline and blank checks, reagent-volume verification, validated temperature and salinity compensation, and periodic comparison with independent reference samples. Continuous-monitoring guidance also emphasizes sensor inspection, calibration verification, and correction of records affected by fouling and drift [94,95].

As discussed in Section 2.3.2, the unified pH concept provides a solvent-independent thermodynamic framework, while the experimental implementations considered here rely on differential potentiometry [34,35,36,37,39]. For the saline-media realization described in that section, the reported expanded uncertainty, *U*(pH) ≈ 0.2, is associated principally with the uncertainty assigned to residual junction effects. This value characterizes the particular experimental realization and its uncertainty budget rather than an intrinsic limitation of the unified pH concept. It should therefore not be generalized to all operational pH scales or applied directly to junction-free spectrophotometric pH_T_ measurements. Although the unified framework offers conceptual advantages for comparing acidity across different media, the cited realization does not yet demonstrate lower uncertainty than established seawater measurement procedures [34,35,36,37,39].

Overall, seawater pH results should identify the pH scale, calibration hierarchy, measurement temperature, salinity, and associated uncertainty. Comparisons among methods require a common reporting basis, documented scale conversions where necessary, and uncertainty budgets appropriate to the analytical procedure and deployment conditions [24,42]. Precision, accuracy, and metrological traceability should be assessed separately, particularly when measurements are used to resolve small environmental changes.

### pH as a Supporting Variable in Emerging Pollutant Monitoring

Continuous pH measurements can support emerging-pollutant monitoring by providing chemical context for contaminant behaviour and sensor response. Changes in pH influence metal speciation and uptake, thereby affecting bioavailability [96]. For ionizable organic contaminants, pH determines the relative proportions of neutral and charged species, which can influence partitioning, bioaccumulation, and toxicity. Experimental studies involving several pharmaceuticals have demonstrated pH-dependent toxicity [97], while investigations of fluoxetine have shown changes in partitioning, bioaccumulation, and acute toxicity across different pH conditions [98]. Although these studies primarily concern freshwater systems, they demonstrate the importance of considering pH when interpreting contaminant behaviour; extrapolation to seawater requires consideration of salinity, ionic composition, and the relevant pH scale.

However, pH is a non-specific supporting variable and cannot independently identify an emerging pollutant or determine its concentration. It should therefore be incorporated into multiparameter monitoring strategies alongside conductivity, dissolved oxygen, turbidity, or fluorescence measurements, with appropriate calibration and quality control [95]. Suspected contamination should be investigated using contaminant-specific measurements and confirmed through targeted chemical analysis.

Reliable pH measurements are also relevant to the interpretation of pollutant-sensor responses. For example, electrochemical determination of diclofenac requires evaluation and optimization of pH because it affects sensor performance [99]. Accordingly, pH-dependent responses should be characterized over the intended operating range, and any compensation procedure should be validated for the target analyte and sample matrix. Reporting the pH scale, measurement conditions, calibration procedure, and associated uncertainty strengthens the comparability and interpretation of these measurements.

## 5. Conclusions and Future Trends

pH is a key parameter for quantifying acidity and monitoring acid-base processes in the ocean. Increasing anthropogenic CO_2_ emissions have altered ocean carbonate chemistry, making accurate, comparable, and metrologically traceable seawater pH measurements increasingly important for scientific research, environmental monitoring, and the assessment of ocean acidification.

This review critically compared five pH scales used or proposed for seawater measurements: the IUPAC, free-proton, total-proton, seawater, and unified scales. The IUPAC pH scale remains the thermodynamic reference and provides the established route to primary pH standards; however, its direct application to seawater is limited by the high ionic strength and complex composition of the medium. Concentration-based scales were developed to address these practical limitations, and the total hydrogen-ion scale, pH_T_, is currently the dominant operational framework for seawater measurements.

The unified pH scale provides a conceptually attractive, solvent-independent framework based on proton chemical potential and linked to the conventional IUPAC pH scale. However, the available evidence indicates that its current differential-potentiometric realization has not yet demonstrated a practical metrological advantage for seawater measurements. Its application to saline media remains strongly affected by residual liquid-junction potentials, resulting in uncertainties substantially larger than those achieved using established seawater pH methods. The unified scale should therefore be regarded as a promising research framework rather than a mature alternative for routine oceanographic monitoring.

For seawater sensor applications, spectrophotometric pH_T_ methods currently provide the most robust route to climate-quality measurements when indicator purity, dye-induced perturbation, optical performance, temperature, salinity, and sample handling are adequately controlled. Potentiometric glass electrodes remain useful for laboratory measurements and short deployments but require matrix-matched calibration and rigorous control of liquid-junction and reference-electrode effects. ISFET, optode, metal-oxide, digital-colorimetric, and microfluidic platforms expand the available options for rapid, autonomous, low-power, or spatially distributed monitoring. Nevertheless, their calibration, drift, biofouling, temperature and salinity cross-sensitivity, matrix dependence, and long-term operational stability require further validation against appropriate seawater reference measurements.

Future research on potentiometric methods should prioritize the development of salt-bridge materials and junction configurations capable of minimizing residual liquid-junction potentials in high-ionic-strength media. Further investigation of ionic liquids, electrolyte combinations, and reproducible junction geometries may improve the differential-potentiometric realization of pH_abs_. These developments should be validated systematically over relevant ranges of salinity, temperature, pressure, and seawater composition and supported by complete uncertainty budgets. Improvements should be evaluated not only through lower measurement uncertainty but also in terms of robustness, traceability, operational feasibility, and long-term field performance.

For spectrophotometric methods, future developments should address indicator characterization and stability, minimization or correction of sample perturbation, reagent-delivery reproducibility, and improvements in optical calibration and long-term stability. Compact systems with reduced reagent and power consumption could facilitate deployment on autonomous platforms while preserving the measurement quality required for long-term ocean-acidification monitoring. Emerging technologies should likewise be assessed under realistic marine conditions and over deployment periods sufficiently long to characterize drift, fouling, sensor ageing, and maintenance requirements.

Harmonization of calibration, validation, and data-reporting procedures is another important priority. Interlaboratory comparisons involving different sensing principles and operational pH scales, together with wider access to characterized seawater reference materials and independently validated reference procedures, would facilitate the identification of systematic differences and improve data comparability. Particular attention should be given to distinguishing accuracy, precision, stability, bias, and measurement uncertainty. Reported performance should be supported by a clearly defined measurand, measurement model, traceability chain, validation procedure, and uncertainty budget.

Ultimately, progress in seawater pH sensing will depend on integrating advances in sensor design, autonomous operation, and data processing with rigorous metrological validation. Future performance should be assessed at the level of the complete measurement system rather than attributed solely to the selected pH scale or sensing principle. The development of reliable and interoperable systems capable of providing traceable data over extended deployments remains essential for detecting and interpreting long-term changes in ocean carbonate chemistry.

## Figures and Tables

**Table 1:** Analytical pH scales used for seawater, representative values, and suitability for in situ monitoring. Values are illustrative and do not constitute a common sample intercomparison. The ${\mathrm{pH}}_{\mathrm{abs}}^{H_{2}O}$ value marked with an asterisk was obtained for an OSIL standard-seawater sample (adapted from [52]).

Concept	Analytical Methods	pH	In Situ Monitoring Suitability	Ref
pH = −lg *a*_H+_	Potentiometry with Harned cell to assign pH to IUPAC buffers and potentiometry with IUPAC buffers	8.33	Limited for direct seawater use; calibration transfer and LJP must be controlled.	[44,45,47,48]
pH_F_ = −lg [H^+^]	Potentiometry using TRIS buffers as a reference with pH assigned by Harned cell	8.20	Used in marine thermodynamic calculations but uncommon for autonomous sensor reporting.	[52,53,60]
pH_T_ = −lg ([H^+^] + [HSO_4_^−^])	Potentiometry using TRIS buffers as a reference with pH assigned by Harned cell and spectrophotometry with indicator dye	8.09	High suitability; the dominant operational scale for ocean-acidification observations and spectrophotometric systems.	[9,10,22,28,31,56,66]
pH_SWS_ = −lg ([H^+^] + [HSO_4_^−^] + [HF])	Potentiometry using TRIS buffers as a reference with pH assigned by Harned cell	8.08	Suitable for defined calculations, but less commonly reported by autonomous instruments than pH_T_.	[52,53]
pHabsH2O=−ΔtrG0H+,H2O →SRT ln10+pHS	Differential potentiometry with glass electrodes and IUPAC buffers (ILSB)	8.15 *	Not yet suitable for routine seawater monitoring in its current experimental realization because residual junction effects dominate the uncertainty.	[33,34,35,36,37,38,39]

* Value obtained for a SSW sample from OSIL.

**Table 2:** Comparison of potentiometric and spectrophotometric platforms for seawater pH measurements. Reported performance values are specific to the methods and conditions in the cited studies; they are not universal measurement uncertainty limits.

Sensing Platform	Calibration and pH Scale	Reported Performance and Uncertainty	Response Time	Stability and Drift	Reagent Consumption	Suitability for Long-Term Monitoring	Ref.
Combination glass electrode	IUPAC buffers, with matrix-matched seawater TRIS calibration or quality control. The reported scale depends on the procedure.	Measurement uncertainty is procedure-specific and requires a complete budget. Principal contributions include buffer values, calibration regression, liquid-junction potential, reference electrode, temperature and salinity mismatch.	Rapid measurement after electrode equilibration; the time depends on the electrode and conditions.	Electrode ageing, reference-electrode changes and junction clogging can cause drift; frequent calibration checks are needed.	None during measurement; calibration and quality-control solutions are required.	Possible with regular calibration and maintenance, but reference stability, pressure effects and biofouling constrain unattended use.	[10,22,23,24,25,26,27,44,45,46,47,48,49,50,51,52,53,54,55,56,77,79,80]
ISFET/DuraFET	Characterized seawater TRIS buffers and/or discrete spectrophotometric reference samples; usually reported as pH_T_.	Accuracy of approximately 0.01 pH units or better and stability of about ±0.005 have been reported under controlled conditions. Field offsets can exceed ±0.03 when calibration or operation is inadequate. These figures are not expanded measurement uncertainties.	Rapid response, suitable for high-frequency observations; the time depends on conditioning and instrument configuration.	Reference-system changes, calibration drift, temperature and salinity effects, pressure response and biofouling require monitoring.	No indicator reagent; calibration and reference samples are required.	Well-suited to autonomous monitoring when reference stability, calibration checks and antifouling measures are maintained.	[81,82]
Discrete spectrophotometric pH_T_	Purified, characterized meta-cresol purple (mCP), with established indicator models and controlled temperature and salinity.	Approximately 0.003–0.005 pH units under controlled conditions, reported as accuracy, intercomparison agreement or practical uncertainty depending on the study. Contributions include indicator properties, absorbance, temperature, salinity, blank correction, mixing and dye perturbation.	Determined by sample handling, indicator addition, mixing and optical acquisition; method-dependent.	Optical and indicator quality must be controlled; long-term instrument drift is generally less relevant to individual discrete measurements.	Indicator is consumed for each sample.	Particularly suitable as a reference or validation method; continuous unattended use requires an automated implementation.	[20,28,29,43,65,66,85]
Autonomous spectrophotometric analyser	Instrument-specific optical and fluidic calibration; validation against discrete purified-mCP measurements or reference materials; normally pH_T_	Published performance is commonly in the low 0.001–0.01 pH range, depending on instrument and validation design. The reported metric may be precision, accuracy, repeatability or difference from a reference, rather than complete uncertainty.	Determined by the sampling cycle, reagent delivery, mixing and optical measurement; instrument-dependent.	Reagent ageing, optical fouling and changes in pumps, valves or fluidic delivery can introduce drift; periodic reference validation is needed,	Indicator reagent is consumed at each measurement; demand depends on sample volume and measurement frequency.	Suitable for high-frequency autonomous observations, subject to reagent reservoir capacity, power, fouling, pump reliability and periodic validation.	[20,78,90,94]

Note: Numerical values in Table 2 reproduce the performance quantities stated in the cited literature. Accuracy, precision, stability, repeatability, bias, deviation from a reference method, standard uncertainty, and expanded uncertainty are not interchangeable. A complete uncertainty statement requires a defined measurand, measurement model, coverage factor where applicable, and evaluation of all relevant calibration and measurement contributions [92,93].

## Data Availability

No primary research results, software or code have been included, and no new data were generated or analyzed as part of this review.

## Abbreviations

The following abbreviations are used in this manuscript:

ASW	Artificial Seawater
EMPIR	European Metrology Programme for Innovation and Research
GOA-ON	Global Ocean Acidification Observing Network
IAPSO	International Association for the Physical Sciences of the Oceans
IL	Ionic Liquid
ILSB	Ionic Liquid Salt Bridge
IPCC	Intergovernmental Panel on Climate Change
ISFET	Ion-Sensitive Field Effect Transistor
IUPAC	International Union of Pure and Applied Chemistry
LJP	Liquid-Junction Potential
NMI	National Metrology Institute
OSIL	Ocean Scientific International Laboratory
RLJP	Residual Liquid-Junction Potential
SB	Salt Bridge
SI	International System of Units
SSW	Standard Seawater
TRIS	Tris(hydroxymethyl)aminomethane

## Nomenclature

The following symbols are used in this manuscript:

${\mathrm{pH}}_{\mathrm{abs}}^{H_{2}O}$	pH in the unified scale anchored to water.
[N_2225_][NTf_2_]	TriethylpentylammoniumBis(trifluoromethanesulfonyl)imide.
*a*	Molal ionic activity, in moles per kilogram of solvent (mol·kg^−1^)
*b*	Molality, expressed in moles per kilogram of solvent (mol·kg^−1^)
*b*^0^	Standard molality (equal to mol·kg^−1^).
*E*	Measured potential between the Standard Hydrogen electrode and silver/silver chloride electrode corrected to a pressure of H_2_ equal to 1 atm (V)
*E*’	Potential of silver-silver chloride electrode in saline medium at a given chloride molality (>0 mol·kg^−1^) (V)
*E*^0^	Standard potential of silver-silver chloride electrode in HCl 0.01 mol kg^−1^ (V)
*E*^0^***	Standard potential of silver-silver chloride electrode in ASW medium(V)
*E*_n_	normalized error
*F*	Faraday constant (exactly 96,485.33212 C·mol^−1^).
*I*	Ionic strength (mol·kg^−1^)
*I*_mean_	average of the intercepts obtained during glass electrodes calibration.
*k*	coverage factor
*K*_B_	Dissociation constant of boric acid.
*K*_i_	Dissociation constant for reaction i.
*K*_W_	Sel-dissociation constant of water.
*p*	Partial pressure in Pa
*p*^0^	Standard atmospheric pressure (101.325 kPa)
*p*CO_2_	Partial pressure of CO_2_ (Pa).
pH_abs_	pH in the unified scale.
pH*_b_*	molal pH scale
pH_F_	Operational definition of the free hydrogen pH scale
pH_IUPAC_	IUPAC pH scale (equal to the negative decimal logarithm of the molal activity of the H^+^ ion).
pH_SWS_	Operational definition of the seawater pH scale
pH_T_	Operational definition of the total hydrogen pH scale
*R*	Molar gas constant (exactly 8.3144626 J mol^−1^·K^−1^).
*S*	Nominal practical salinity
*S*_A_	Absolute salinity (g·kg^−1^)
*s*_mean_	average of the slopes obtained during glass electrodes calibration.
*T*	Temperature (K or °C)
*u*_x_	Standard uncertainty
*U*_x_	Expanded uncertainty (*k* = 2).
Δ_solv_*G*^0^	Gibbs standard solvation free energy (kJ·mol^−1^).
Δ_tr_*G*^0^	Gibbs energy of transfer (kJ·mol^−1^).
ν	Amount content, expressed in moles per kilogram of solution (mol·kg^−1^)
*γ*_Cl−_	Activity coefficient of Cl^−^.
*γ*_HCl_	Mean ionic activity coefficient of HCl.
*γ*_H+_	Activity coefficient of H^+^.
*μ*^0^_abs_ (H^+^)	Absolute standard chemical potential of the proton (kJ·mol^−1^).
*μ*_abs_ (H^+^)	Absolute chemical potential of the proton (kJ·mol^−1^).
*ρ*	Density in kilogram per cubic decimeter (kg·dm^−3^)
*ω*_H2O_	Water mass fraction
